# Reviews and Thoughts on the Relevance between Qualitative Chinese Medicinal Properties and Quantitative Material Components

**DOI:** 10.1155/2020/8643746

**Published:** 2020-07-14

**Authors:** Zheng-Kun Hou, Wen Hu, Feng-bin Liu, Ying-yu Liang, Zhong-yu Huang, Zhi-bang Huang

**Affiliations:** ^1^Department of Gastroenterology, First Affiliated Hospital, Guangzhou University of Chinese Medicine, Guangzhou 510405, Guangdong, China; ^2^Intensive Care Unit, Huanggang Hospital of Traditional Chinese Medicine, Huanggang 438000, Hubei, China; ^3^College of Traditional Chinese Medicine, Guangdong Pharmaceutical University, Guangzhou 510006, Guangdong, China; ^4^Integrated Chinese and Western Medicine Postdoctoral Research Station, Jinan University, Guangzhou 510632, Guangdong, China; ^5^Graduate College, Guangzhou University of Chinese Medicine, Guangzhou 510006, Guangdong, China

## Abstract

**Objective:**

Chinese Medicinal Properties (CMP) play a vital role in theoretical research and clinical practice. However, the traditional CMP system is subjective, qualitative, fixed, inconsistent, and obscured. Nowadays, quantifying CMP research achieved a notable progress. This study aims to review and reflect the relevance between qualitative CMP and quantitative material components.

**Methods:**

A raw literature search was performed firstly in CNKI and Pubmed database to get a rough idea on the general advances in measuring CMP. Then, a strict literature search and data extraction from two dependent research studies were performed to analyze the relevance and discrimination between CMP and material components.

**Results:**

The quantitative CMP research mainly focused on the microelements and chemical compositions. The largest microelements research listed 747 Chinese Materia Medica (CMM) (6780 flavors) and 120,000 element data. The measurement of chemical composition of CMM has risen rapidly in the 1990s and continues till the present. Thirty-seven articles were finally identified for the relevance analysis of CMP and material components. Of these, 18 and 19 articles correspondingly focused on the chemical compositions and microelements, and 26 and 11 articles correspondingly focused on their correlation and discrimination relationship. The most commonly used method for correlation analysis is intuitive analysis. The support vector machine maybe highly efficient and would act as the preferred method in discriminant analysis. Twelve (67%) and 5 (26%) articles' data came from the literature search in chemical compositions and microelement research studies. Four studies indicated that the research objects are the basic substances and material basis of CMP, 15 articles claimed that the chemical compositions were significantly related to CMP, 12 research studies concluded that the regularity and causality were identified between the research objects and CMP, and 9 research studies successfully established discriminant models for CMP basing on the detected substances.

**Conclusions:**

The relevance research between qualitative CMP and quantitative material components achieved a positive progress, though it is weak and defective. Standardizing the qualitative CMP system, establishing series comprehensive databases for the material components, innovating statistical and data mining methods, and integrating doctors' experiences are important and feasible for future research.

## 1. Introduction

The medicinal property (or nature, categorization) is the basis of prescription and efficacy in Traditional Chinese Medicine (TCM), which plays a vital role in theoretical research and clinical practice. It was established and enriched by many highly skilled and respectable ancient Chinese physicians and pharmacists in the last thousands of years. Nowadays, the Chinese Medicinal Properties (CMP) system contains four Qi (or four natures, si qi), five flavors (wu wei), ascending, descending, floating and sinking (sheng jiang fu chen), channel entering (or meridians, gui jing), and toxicity [1]. This system has made great contribution to classify Chinese Materia Medica (CMM), activate the efficacy of drugs and formulas, heal body, and even evaluate and diagnose diseases.

However, the current CMP system has obvious shortcomings. It is (a) subjective, that is, artificially classified by ancient physicians and pharmacists just based on their own feelings or experience in clinical practice; (b) qualitative, that is, described only with text rather than data; (3) fixed, that is, rarely changed after proposed by the original person; (4) inconsistent, that is, the medicinal properties for some herbs were described differently in different medical books; and (5) the latent characteristics of medicinal properties are unknown due to outdated natural techniques in ancient times. These limitations obviously hinder the communication of TCM and CMM, both internally and externally to Western medicine, as well as global doctors and researchers and, secondly, decrease the technical capabilities of clinicians, especially for beginners and integrative doctors who need to devote more energies to realize and think about these vague drug characteristics.

It is, however, gratifying that these limitations may not be insurmountable. Quantifying the properties of CMM is one important and feasible method. CMM quantification is a scientific, standardized, specific, micro, and quantifiable modern research category, which contains dose thresholds from a scientific view and syndrome differentiation and dose from philosophical and artistic views [2]. Of those, quantifying CMP is the core set which intends to measure, standardize, apply, and visualize the latent natures and representative characterizations of CMM. It transforms the traditional qualitative CMP system into the modern quantitative one. This study aims to analyze the advances and limitations of the current quantitative CMP research and provide advice for future research.

## 2. The General Advances in Measuring CMP

The material components of CMM are mainly divided into inorganic, organic, and physical compositions. In the <Chemistry of Chinese Materia Medica>, it was divided into more detailed categories: sugars, glycosides, terpenoids, phenylpropanoids, flavonoids, steroids and volatile oils, alkaloids, steroids, triterpenoids, and tannins, as well as fatty acids, organic phosphorus compounds, amino acids, cyclic peptides, proteins, enzymes, and minerals [3]. Currently, the quantitative CMP research mainly focused on the compositions measurement of CMM, which has made significant strides with the rapidly developing microscopic measurement technologies, especially in the last decades.

**Figure 1:**
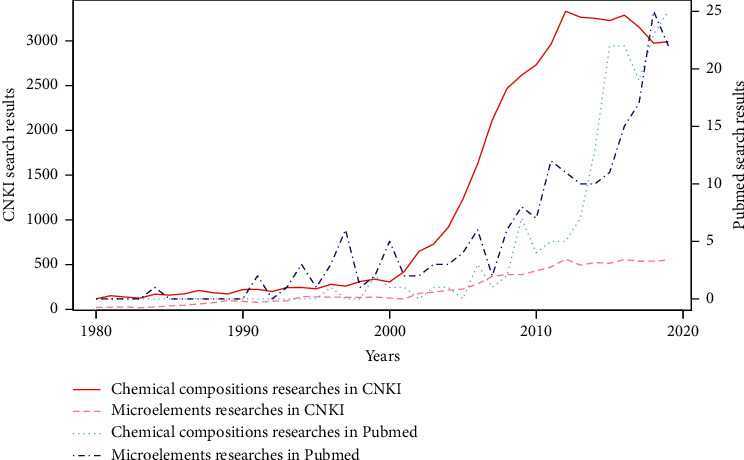
The raw search of general advances of measuring Chinese medicinal properties in CNKI and Pubmed database^*∗*^. ^*∗*^Search strategy in CNKI: common words: AB = (“si qi”+“wu wei”+“gui jing'+“sheng jiang fu chen”+“du xing”+“yao xing”+“gong xiao”+“xin”+“ku”+“gan”+“xian”+“ping”+“han”+“re”+“wen”+“liang”+“suan”); for Microelements researches, add: AND AB=(“yuan su”+“wei liang yuan su”); for Chemical Compositions researches, add: AND AB=(“you ji hua xue”+“you xiao cheng fen”+“hua xue cheng fen”+“huo xing wu zhi”+“gan lei”+“tang lei”+“kun lei”+“huang tong lei”+“hui fa you”+“tie lei”+“sheng wu jian”+“zai ti lei”+“san tie lei”+“rou lei”); Discipline Category: Medicine; Last retrieval time: Dec. 24, 2019; Search strategy in Pubmed: (Four Qi[Title/Abstract] OR Five Flavors[Title/Abstract] OR channel entering[Title/Abstract] OR properties[Title/Abstract] OR nature[Title/Abstract]) AND ((Chinese Medicine) OR (Chinese Medicinal) OR (Chinese Materia Medica) OR (Chinese herbs) OR (Chinese herbal)); for Microelements researches, add: AND (Elements[Title/Abstract] OR Microelement[Title/Abstract] OR Trace elements[Title/Abstract]); for Chemical Compositions researches, add: AND (Organic Chemistry[Title/Abstract] OR Active Ingredients[Title/Abstract] OR Chemical Ingredients[Title/Abstract] OR Active. Substances[Title/Abstract]); last retrieval time: Dec. 24, 2019.

Since the 1960s, a large number of researchers have studied the active ingredients and basic substances of CMM. In 1973, Nozdryukhina discovered the biological activity efficacy of microelements in Chinese herbs [4]. This finding has greatly influenced the microresearch of CMM during the later decade, and plenty evidence were accumulated. In 2013, Li et al. [5] published series articles listed 747 CMM (6780 flavors) and 120,000 element data which came from 1036 literatures and 34 kinds of analytical technologies. Since the 1990s, with the development of chemical compositions detection technologies in China, the measurement of chemical compositions of CMM has risen rapidly and continues till the present. This research category has achieved greater progresses and accumulated more evidences than microelements research (Figure 1). However, as for the published articles in Pubmed, the difference is not so obvious, but the total number (no more than 25 per year) is significantly smaller than the articles in CNKI. Besides many professional papers, journals, and books, many chemical databases and TCM databases provided detailed information on the chemical compositions of CMM.

## 3. The Advances in the Relevance and Discrimination between CMP and Chemical Components

### 3.1. Search Strategy

The searches were performed in the CNKI and Pubmed database with predefined search strategies: “SU=(“si qi”+“wu wei”+“gui jing”+“sheng jiang fu chen”+“du xing”+“yao xing”+“gong xiao”+“xin”+“ ku”+“gan”+“xian”+“ping”+“han”+“ re”+“wen”+“liang”+ “suan”) *∗* (“liang hua”+“ding liang”+“pan bie”+“guan xi”+“guan lian”+“xiang guan”) AND AB=(“yuan su”+“wei liang yuan su”+“you ji hua xue”+“you xiao cheng fen”+“hua xue cheng fen”+“huo xing wu zhi”+“gan lei”+“tang lei”+“kun lei”+“huang tong lei”+“hui fa you”+“tie lei”+“sheng wu jian”+“zai ti lei”+“san tie lei”+“rou lei”)” limited in “Medicine” discipline category in CNKI database; “(((Quantitative[Title] OR Discrimination[Title] OR Relationship[Title])) AND (Elements[Title/Abstract] OR Microelement[Title/Abstract] OR Trace elements[Title/Abstract] OR Organic Chemistry[Title/Abstract] OR Active Ingredients[Title/Abstract] OR Chemical Ingredients[Title/Abstract] OR Active Substances[Title/Abstract])) AND (Four Qi[Title/Abstract] OR Five Flavors[Title/Abstract] OR channel entering[Title/Abstract] OR properties[Title/Abstract] OR nature[Title/Abstract])” in Pubmed database. The last retrieval time is Dec. 24, 2019.

### 3.2. Data Management and Extraction

The literature was included if it met all the following criteria: (a) analyzed the relevance or discrimination between CMP and material components; (b) adopted clear statistical methods or tools.

The literature was excluded if it met any of the following criteria: (a) only detected chemical components of CMM without relevance or discrimination analysis; (b) qualitative interpretations, hypotheses, speculations, or reviews of CMP without quantitative evidences; (c) duplicated publications; and (d) full text was not available.

Two researchers (HU Wen, HUANG Zhi-bang) independently searched and traced relevant references. After obtaining the preliminary information, they screened the literature according to the predefined inclusion and exclusion criteria. If the results are inconsistent, they presented them to the coordinators (HOU Zheng-kun, LIU Feng-bin) for further evaluation until an agreement was reached. Then, HU and HUANG independently extracted information from the literature with a uniform data form. HOU checked the final data and retracked and re-extracted the information if a difference or bias was perceived.

### 3.3. Main Results

#### 3.3.1. Search Results

**Figure 2:**
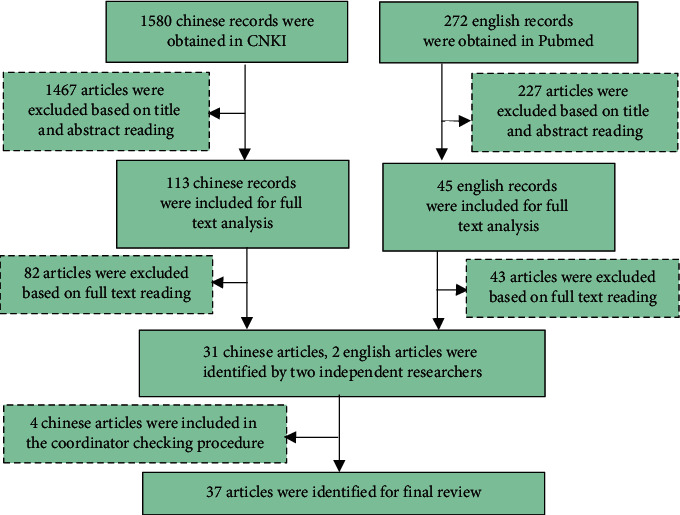
The flow diagram of literature screening for the relevance and discrimination research studies between Chinese medicinal properties and material components in CNKI and Pubmed database.

A total of 1580 and 272 initial records were obtained from CNKI and Pubmed database, respectively. Following the data extraction and management procedure, 35 Chinese documents and 2 English documents were finally included for analysis [6–42] (Figure 2). Of these, 18 [6–23] and 19 [23–42] articles respectively focused on the chemical compositions and microelements to analyze the correlation or discrimination with CMP (Tables 1 and 2).

#### 3.3.2. Correlation, Discrimination, Quantitative Research, and Methods

Firstly, there are 26 articles focused on the correlation relationships between CMP and chemical compositions [6–9, 11, 14, 16–20, 22, 23, 37] or microelements [24–29, 31, 33–36, 39]. Among them, the most commonly used method is intuitive analysis [6, 7, 9, 17, 33, 34, 37], that is, the researcher investigates the macroscopic drug properties from a microscopic angle by synthesizing from the numeric data to find the correlations between microscopic substances and CMP. The other analysis methods include factor analysis, cluster analysis, logarithm, standard error, *t*-test, variance, and correlation analysis, which also aim to find the correlations between the chemical components and CMP based on the composition and content differences of CMM with different medicinal properties.

Secondly, there are 11 articles in which the discrimination relationships between CMP and chemical compositions [10, 12, 13, 15, 21] or microelements [30, 32, 38, 40–42] are studied. The research methods used include cluster analysis, principal component analysis, discriminant function, Fisher analysis, Bayes discriminant analysis, support vector machine, and so on. It is notable that Yang [10] established a logistic regression equation for four levels of organic components and identified the relationship between inorganic elements and cold-heat of herbs. The results confirmed that the high Fe and low Mn content are the character of cold CMM and low Fe and high Mn content are the character of hot CMM. In the prediction and discrimination research on the 80 CMM properties, basing on the types and contents of inorganic elements, the accuracy rates of Fisher, Bayes, and support vector machine methods were 60%, 78.75%, and 95%, correspondingly. Qin et al. [15] established a support vector machine discriminant model based on the volatile components of 28 antiepidemic drugs, and the accuracy rate was 93.6%. The limited evidence showed that the support vector machine maybe highly efficient and would act as the preferred method in discriminant analysis. In addition, Hu et al. [28] analyzed the relationship between four characteristics and 32 kinds of trace elements in 115 Chinese herbs with linear discriminant analysis and established a model with 70.34% correct classification for cool, warm, and neutral. Liang [37] proposed that the four Qi and five flavors should be quantified with general acid-base theory, the principle of soft-hard and acid-base, Frontline orbit theory, and the relationship between electronegativity difference and ionic properties of the chemical bond.

#### 3.3.3. Data Source of Chemical Composition of CMP

Of the 18 articles which analyzed chemically active ingredients, 12 (67%) research studies' data came from literature search [6–10, 13, 14, 16–18, 20, 21], and only 6 (33%) research studies's data came from self-test experiments [11, 12, 15, 19, 22, 23]. Of the 19 articles which analyzed microelements, the number is 5 (26%) [24–26, 34, 40] and 14 (74%), correspondingly, but the tests mainly concentrated in the 1990s and early 2000s.

#### 3.3.4. Main Findings and Conclusions of Research Studies

The abovementioned quantitative research explores the CMP from multiple angles, such as chemical active ingredients, microelements, protein content, molecular charge, molecular weight, and so on. Four literatures indicated that the research objects are the basic substances and material basis of nonrepresentational CMP [12, 28, 35, 39], 15 articles claimed that the chemical compositions of CMM were significantly related to the CMP [6, 8–11, 14, 15, 20, 22, 23, 26, 27, 30, 32, 37], and 12 researches concluded that the regularity and causality existed between the research objects and CMP [7, 16–19, 24, 25, 29, 31, 33, 34, 36]. For example, Liu et al. [16] found that the CMM with diuresis efficacy which attributed in upper Jiao (上焦) major contain flavonoids and followed with terpenoids, in middle Jiao (中焦) major contain terpenoids and followed with steroidal, and in lower Jiao (下焦) major contain terpenoids and followed with flavonoids. Jia et al. [18] pointed out that composite Chinese Medicinals mainly owned the nature of being cold and cool and mainly contain volatile oils, flavonoids, terpenes, alkaloids, and acids, which are closely associated with their efficacy. Gong and Zhang proposed that the contents of Fe, Zn, and Cu are higher in CMM with cold property and salty flavor [24] and Zn, Fe, Cu, and Mn contents are higher in CMM which enter the liver channel [25]. Also, 9 research studies established discriminant function models for CMP, which can successfully discriminate CMP based on the detected substances [10, 13, 15, 21, 30, 32, 38, 40, 41].

## 4. Discussion

As mentioned above, a number of research studies have been performed on the measuring of material components of CMM, conversely, less focused on the relationships between the material components and CMP. In terms of data sources, many studies abandoned self-test but adopt literature reviews. Even so, many literatures did not strictly define the data sources and inclusion and exclusion criteria. As for the analysis methods, the earlier studies usually performed analysis with basic statistical methods and indicators, such as frequency, logarithm, mean, standard error, standard deviation, and so on; now, more and more studies use complex methods, such as factor analysis, cluster analysis, support vector machine, and Fisher analysis, that is, some progress in methodologies has been achieved. But, more advanced methods, and maybe more suitable for the nonlinear and black-box characters of CMP, such as support vector, neural network analysis, decision tree, and random forest, are still rarely being used. Focusing on the research results, it is clear that the chemical component of CMM is the material basis of CMP, and the regularity and causality do existed between them which can be quantified by discriminant functions.

Another important feature is that the existing quantitative research studies mainly focused on the microelements and chemical compositions of the four Qi (cold, heat) and five flavors (Xin) in CMP, but little on the other microdata and CMP characters, such as channel entering (gui jing) and toxicity, especially, no research was interested in the ascending, descending, and floating and sinking (sheng jiang fu chen). For example, as for the channel entering, Wang et al. [20] found that the CMM in the heart channel contains plenty of terpenoids, flavonoids, and volatile oils, in the liver channel contains plenty of flavonoids, organic acids, and terpenoids, and in the lung channel contains plenty of flavonoids, volatile oils, glycosides, and terpenoids and concluded that there are explicit interrelations of channel tropism of CMM in the chemical constituent. Wang [43] considered the biological basis of different Chinese meridian herbs with network pharmacology and molecular docking methods and found special proteins related to 12 channels. As for the toxicity, Li et al. [44] established a quantitative structure-toxicity model for aconitine compounds basing on partial least squares methods; using the same methods, Xiao et al. [45] established a similar quantitative structure-toxicity model for norditerpenoid alkaloids, and Xin et al. [46] proposed an integrative model for pharmacodynamic research of the aconiti kusnezoffii radix basing on TK-TD from the perspectives of structure-activity and dose effect relationship. As for the processing methods of CMM, Zhong et al. [47] found that different ginger juices will lead to the different changes of the effective material group, for example, jatrorrhizine hydrochloride, coptisine hydrochloride, palmatine hydrochloride, berberine hydrochloride, and epiberberine in Coptidis Rhizoma, resulting in the pharmacological effect and property difference between Coptidis Rhizoma processed with fresh ginger juice and Zingiberis Rhizoma juice.

In addition, some new values and directions had been found by the active researchers. Jing et al. [48] studied the nature-effect relationship of CMM with the main active components, targets, and protein interaction network and found the common characteristics of the bitter-liver combination and the specific characteristics of cold or warm medicinal properties at the molecular network level. Similar research studies were performed to find the nature-effect relationship of Salviae Miltiorrhizae Radix et Rhizomaand and Carthami Flos [49] and Curcumae Longae Rhizoma, Curcumae Radix, and Curcumae Rhizoma [50]. Wang [51] synthesized and analyzed the information on “chemical composition-biological activity-targets” of Acai (Euterpe Oleraceae Mart.) and Maca (Lepidium meyenii Walp.), two new foreign resources which did not exist in the classical CMM contents, and successful predicted and validated their four Qi, five flavors, ascending, descending, floating and sinking, channel entering, and efficacy. Guohui et al. [52] used the Mahalanobis distance learned by distance metric learning algorithm to measure the similarity of ultraviolet spectrum data of the existing 61 herbs and constructed their prediction and recognition model of the cold and hot nature with better predictive stability and extrapolation compared with the existing classical models. Besides, there is one study which explained the substantial base of the nature of Radix Scutellariae through investigating the impacts of the herb and its splitting components on the relevant enzyme expressions of the substance and energy metabolism in rats with cold and heat syndrome and found that aglycones and glycosides are the material base of the cold nature of Radix Scutellariae [53].

However, the current research studies have obvious limitations: firstly, the comprehensiveness and accuracy in the tremendous amount of chemical components data collection procedure directly influence the accuracy and reliability of the experimentation results. For example, the information selection and analysis and import of CMM sources, component categories, and compositions content could produce errors or selective bias and lead to inevitable deviations between the experimental results and true values. More seriously, many studies obtained the data from the secondary literature, 46% from our findings, and subjectively selected some chemical components to perform the analysis without regulation and interpretation. This undetected and unexpressed heterogeneity obviously harms the research credibility. Secondly, the qualities of the research methods used to analyze the relationships between the material components and CMP are various. For example, the subjective analysis method used in many studies is too dependent on the personal experience of experts and lacks credible standards. Using the difference analysis methods, such as standard deviation and *t*-test, it is difficult to solve the complex nonlinearity and diversity problems in the discrimination, prediction, and quantification of CMP. Even for the advanced statistical methods, such as factor analysis, cluster analysis, support vector machine, and Fisher analysis, their working efficacies are also not completely satisfied. Thirdly, there are great gaps between the laboratory researches and clinical practices, that is, all the abovementioned quantitative research studies of CMP are limited in the laboratories and the literature and rarely involved in the clinical services. Unfortunately, the microdata on CMP are not completely consistent with the macrosubjective judgments in real world. The busy physicians still highly rely on their own subjective experiences in clinical practice but not microcomplex objective data. This embarrassing situation makes the quantitative CMP research lack breakthrough and application.

The reasons for the abovementioned limitations are complexly. Firstly, the latent inner properties of CMM are too abstract and indelible, and the chemical components are obviously affected by the region, geology, climate, weather, material extraction time, and processing methods of CMM. Secondly, it is still impossible to accurately measure all the components of CMM with current detection methods, and the results are very susceptible to the technologies and tools. Thirdly, the lack of systematic human, material, and financial investments and managements results in the fragmentation of quantitative CMP research. Fourthly, the lack of multidisciplinary talents has limited the scope of related research, and it is difficult to break into new research fields. However, in any case, these problems are likely to be overcome in the near future.

## 5. Conclusions

In the long course of the history of traditional Chinese medicine, the properties system of Chinese Materia Medica has been established, intermingled, and enriched with the continuous contributions from many ancient doctors and pharmacists, which provides a solid theoretical basis for clinical practice. However, it cannot be evaded that the classical qualitative Chinese medicinal properties system has several deficiencies, which limit the recognition, application, and communication of the traditional Chinese medicine and Chinese Materia Medica. Therefore, measuring, standardizing, applying, and visualizing the Chinese medicinal properties are emphasized and advocated by more and more researchers. Currently, there are many research studies which measured the material components of Chinese Materia Medica and obtained huge information about the microelements and chemical compositions. In addition, there are limited studies which analyzed the relationships between the material components and Chinese medicinal properties. However, there are still many shortcomings in the current research studies, which are mainly related to the complex attributes of Chinese Materia Medica and imperfect measurement techniques, but they are still expected to be resolved. In future research, it is very important and also feasible to standardize the qualitative Chinese medicinal properties system, establish series comprehensive databases for the material components of Chinese Materia Medica, innovate the statistical methods for mining the relationships between the material components and Chinese medicinal properties, and develop a mixed system of Chinese medicinal properties for clinical applications.

## Figures and Tables

**Table 1:** The 18 articles focused on the relevance and discrimination between Chinese medicinal properties and chemical compositions.

First author	Year	Region	Research objects	Research methods	Main results or conclusions	Statistical methods
Chen and Chen [6]	1993	Zhejiang	414 CMM with various flavors and traits	Analyze the material basis and action principle of the functions for five flavors	The five flavors have an inevitable close relationship with the active ingredients of CMM	Intuitive analysis

Hu [7]	1996	Hunan	92 main active ingredients of CMM	Analyze the relationship between the CMP and the molecular weight of major components of CMM	The CMP change regularly with the molecular weight of the main contained active ingredients	Intuitive analysis

Fan and Wang [8]	2008	Beijing	247 vine Chinese medicine data	Correlation analysis of four gas properties and chemical components in Rattan herbs	There is a certain correlation between the four gas properties of Rattan herbs and the chemical constituents and drug efficacy contained in drugs	Correlation analysis

Yao et al. [9]	2008	Chengdu	Herbs for inducing resuscitation	Analyze the working mechanism of volatile components and their correlations with CMP	There is a correlation between the CMP of aromatic herbs and their material basis and pharmacological effects	Intuitive analysis

Yang [10]	2010	Shandong	The chemical compositions of 100 botanicals	Logistic regression equations were established at the four levels of organic composition categories, subcategories, substructural groups, and single compounds to discriminate the cold and heat properties of CMM	There is a correlation between the cold and hot CMP and the chemical constituents of CMM. The discriminative model of cold and hot CMP was established based on the chemical components. The cold and hot CMP can be predicted and discriminated	Logistic regression analysis, rank sum test, principal component analysis, cluster analysis, Fisher analysis, Bayesian analysis, and support vector machine

Shuai et al. [11]	2010	Shandong	The protein content of 50 Chinese herbs determined by the Coomassie brilliant blue method	Measuring the amount of protein in 25 cold, 25-flavored CMM	The average protein content of hot CMM was 2.37 times that of cold CMM, indicating that the protein content had a certain correlation with the cold and hot CMP	*t*-test

Zhou et al. [12]	2010	Shandong	The water-soluble sugars of 10 cold and 10 hot CMM determined by GC/MS fingerprinting	Fisher method to establish discriminant function	Water-soluble sugar is one of the material bases of cold and hot CMP	Fisher method

Long et al. [13]	2011	Tianjin	284 CMM with cold/hot properties and their chemical components	A novel strategy, weight center treatment was used to solve the problem that the chemical description was unable to be applied to CMM	The accuracy of 83.3% and 81.0% for the training and the test set indicate that this system is a useful tool to predict the property of unidentified folk herbs and foreign herbs	Support vector machine, descriptors generation, and weight center calculation

Liang et al. [14]	2013	Beijing	20 CMM with cold (10) and hot (10) properties and their active target proteins and main compounds	The active target proteins were analyzed to find out the property-related biological activities, and the main compounds were analyzed to decipher the properties	Cold-propertied CMM show intensive toxicity in the heart, which is likely to be correlated with the specific chemical fragments constructions, such as long chain alkenes, benzoheterocycle, and azotic heterocycle	Bioinformatics approach

Qin et al. [15]	2013	Guangxi	28 herbs that release the exterior and their volatile ingredients	Cross-train the data with support vector machines and establish a predictive model for the CMP	There is a high correlation between the volatile components and the cold and hot CMP	Support vector machines

Liu et al. [16]	2015	Nanjing	90 CMM with diuresis efficacy	Ancient and modern literature was employed to construct database. Data mining technology was used to analyze the intrinsic relation between CMP and effective components	The CMM attributed in upper Jiao major contain flavonoids and followed with terpenoids, in middle Jiao major contain terpenoids and followed with steroidal, and in lower Jiao major contain terpenoids and followed with flavonoids	Data mining, association rule

Yang et al. [17]	2015	Nanjing	34 kinds of volatile oils with transdermal penetration-promoting effects	Using frequency analysis, variable cross-tabulation for visual analysis and correlation analysis	There are clear correlation and regularity between the transdermal and permeation-promoting effects of essential oils and the CMP	Intuitive analysis, correlation analysis, and mathematical modeling

Jia et al. [18]	2015	Beijing	157 asteraceae CMM and their chemical constituents	Two-factor correlation analysis method	The main chemical constituents contained in cold, warm, and neutral CMM are volatile oils, flavonoids, terpenes, alkaloids, and acids	Correlation

Jiang et al. [19]	2016	Nanjing	18 kinds of volatile oil	Logistic regression analysis	There is a clear correlation and regularity between the CMP and the composition of volatile oils	Logistic regression analysis

Wang et al. [20]	2018	Shenyang	416 CMM attributed to heart, liver, and lung channel and their chemical constituent, pharmacological effect, and clinical application	Frequency analysis with 5% as the cut point. The chemical constituents, pharmacological effects, and clinical applications were analyzed with association methods, and the results were selected with support degree >5% and confidence >40%	It has explicit interrelation of channel tropism traditional Chinese medicine in chemical constituent, pharmacological effects, and clinical application	Frequency statistics, corresponding analysis, and association rules

Jiang et al. [21]	2019	Shenyang	11 Yang-tonifying herbs	The empirical regression equation was constructed to explore the tissue distribution of the receptors in the training set, and the criterion for determining whether herbs distribute to kidney meridian was established	This study explored a new method for judging whether CMM distributes to kidney meridian, established an effective criterion model, and verified the reliability of the new method	Empirical regression, neural network

Wenguo et al. [22]	2019	Nanjing	20 kinds of essential oils with different “four natures” drug properties	Essential oils were extracted by steam distillation and analyzed by GC-MS, and the skin resistance kinetic technology was used to investigate the abilities of penetration enhancement. The factors were selected by the stepwise discrimination and variance analysis method	There was a correlation among the “four natures” drug properties, the abilities of penetration enhancement, and chemical components of essential oils from pungent Chinese herbs	Stepwise discrimination analysis method, and variance analysis method

Dai and Xue [23]	2019	Leshan	9 kinds of essential oils with different “four natures” drug properties	The volatility method was used to analyze the different volatile oil components. The relationship between volatile oil and CMP was analyzed by logistic regression analysis	The different types of volatile oils are related to the four natures and five flavors of CMP. The transdermal penetration promoting effect of volatile oil has certain correlation and regularity	Logistic regression

**Table 2:** The 19 articles focused on the relevance and discrimination between Chinese medicinal properties and microelements.

First author	Year	Region	Research objects	Research methods	Main results or conclusions	Statistical methods
Gong and Zhang [24]	1990	Yangzhou	182 CMM and their microelements content	Analyze the microelements content by logarithm, logarithm mean, standard error, and standard deviation	High levels of Fe, Zn, and Cu in cold, cool, and salty Chinese herbs	Logarithm, logarithmic mean, standard error, and standard deviation
Gong and Zhang [25]	1990	Yangzhou	182 CMM and their microelements content	Analyze the microelements content by logarithm, logarithm mean, standard error, and standard deviation	The CMM attributed in the liver channel are rich in Fe, Zn, Cu, and Mn	Logarithm, logarithmic mean, standard error, and standard deviation
Chen [26]	1990	Jiangxi	176 CMM and the Fe, Zn, Cu, and Mn contents	Analyze the relationship between the content and ratio of each elements and CMP	The CMP are closely related to the contents of iron and manganese	Linear correlation and regression analysis
Chen et al. [27]	1992	Jiangxi	18 CMM and their 15 microelements content	Analyze the relationship between the content and ratio of each elements and CMP	The CMP are related to the different contents of certain microelements	Variance analysis
Hu et al. [28]	1992	Shandong	32 CMM (115 flavors)	Establish the discriminant equations in three types of drugs (homothermic drugs, flat drugs, and herbal drugs)	The content of inorganic elements is the material basis that determine the four properties of CMP	Linear discriminant analysis
Tang and Guan [29]	1994	Wuhan	27 CMM and 15 rare Earth elements and 27 nonrare Earth elements content	The significance of the difference in the contents of 42 elements in simulant, sweet, and bitter CMP was examined by the *t*-test	Most elemental contents in pungent CMM are higher than sweet and bitterness CMM	*t*-test
Xue [30]	1996	Wuhan	5 CMM and their 19 elements content	Two kinds of sample homogeneity of the variance test and two kinds of discriminant analysis were used	Establishing the discriminant equation for CMM efficacy	Variance homogeneity test and the discriminant function method
Hong et al. [31]	1996	Jiangxi	7 Yang-tonifying herbs and their 15 vital elements content	Analyze the average content and standard deviation of each element of various drugs	High in Zn, Mn, Cu, Fe, and Cr elements and low in Pb, Cd, and Ba are the first choice for Yang-tonifying herbs	Standard deviation
Chen et al. [32]	1996	Jiangxi	100 CMM, and their 15 inorganic element content	Stepwise discriminant analysis was used to analyze the relationship between 15 elemental contents and drug properties	Establishing the discriminant function of inorganic chemical elements for CMP	Discriminant function method
Qin et al. [33]	1998	Liaoning	16 herbs that tonify the body and their 11 microelements content	Analyze the pharmacology and correlation of effects of inorganic chemical elements in CMM	Microelements have the tonic effect	Intuitive analysis
Wang et al. [34]	2001	Beijing	14 kinds of tonic herbs and their microelements content	Analyze the pharmacology and correlation of effects of inorganic chemical elements in CMM	The content of microelements in herbs that tonify the blood is greater than herbs that tonify the Qi	Intuitive analysis
Qi et al. [35]	2003	Chongqing	10 herbs that release the exterior and their 16 microelements content	Factor analysis and cluster analysis were used to analyze the characters of 16 microelements in 10 CMM	The content of inorganic elements are the material basis that determine the CMP	Factor analysis and cluster analysis
Jin and Yan [36]	2003	Beijing	A large number of Chinese herbal elements in the literature	The statistical parameters of the distribution of microelements in CMM	There is a causal relationship between Yin/Yang CMP, flavors, organic components, and microelements in CMM	Cluster statistics
Liang [37]	2004	Nanjing	12 mineral CMM and the active ingredients	Quantifying according to the generalized pH theory, soft and hard acid and alkali principles, frontier orbit theory, relationship between electronegativity differences and key ionicity, and Pka values	Proposed three ways to quantify four flavors and five flavors	Intuitive analysis
Ma and Guan [38]	2004	Wuhan	105 CMM and their 42 elements content	Using Wilks'*λ* minimization method to screen 11 elements that significantly contribute to drug taste and establish an “optimal” linear discriminant function according to the Bayes criterion	Establishing three discriminative functional formulae for pungent, sweetness, and bitterness	Wilks' minimization method
Zhao et al. [39]	2007	Beijing	8 CMM and their 11 elements content	Using the calculated cluster parameters to deeply analyze the taste and pharmacological activities	According to the subparameters, 8 anti-AIDS drugs were divided into three categories: drug-induced partial sun, partial vaginal Yin, and Yin-Yang positive drug	Cluster statistics method
Liu et al. [40]	2009	Sichuan	193 CMM and their 7 elements content	Using support vector machines to train 193 CMM and establish a predictive model for neutral and nonneutral CMP	There is certain correlation between the content of inorganic elements and CMP	Support vector machines
Wu et al. [41]	2010	Wuhan	105 CMM and their 42 elements content	Establishment of the Fisher discriminant equation based on the content of 42 microelements in 105 CMM	Establish the Fisher discriminant equation	Fisher discriminant analysis
Wu et al. [42]	2012	Wuhan	105 CMM and their 42 elements content	After the Fisher discriminant analysis, equations are established and evaluated	The established discriminant equation is active and useful	Fisher discriminant analysis
